# Analysis of Earlier Temporomandibular Joint Disorders in JIA Patients: A Clinical Report

**DOI:** 10.3390/healthcare9091140

**Published:** 2021-08-31

**Authors:** Alessandro Polizzi, Vincenzo Quinzi, Simona Santonocito, Giuseppe Palazzo, Giuseppe Marzo, Gaetano Isola

**Affiliations:** 1 Department of General Surgery and Surgical-Medical Specialties, School of Dentistry, University of Catania, Via S. Sofia 78, 95124 Catania, Italy; alexpoli345@gmail.com (A.P.); simonasantonocito.93@gmail.com (S.S.); gpalazzo@unict.it (G.P.); 2 Department of Life Health and Environmental Sciences, University of L’Aquila, 61700 L’Aquila, Italy; vincenzo.quinzi@univaq.it (V.Q.); giuseppe.marzo@univaq.it (G.M.)

**Keywords:** temporomandibular joint, juvenile idiopathic arthritis, temporomandibular dysfunction, mastication, quality of life, clinical trial

## Abstract

The aim of this study was to analyse the structural characteristics of the temporo-mandibular joint (TMJ) and the dysfunctional consequences induced by disease in subjects with juvenile idiopathic arthritis (JIA). The study was conducted in 25 patients with JIA (median age (IQR), 13.9 (10.9–15.3)) and 26 healthy controls (median age (IQR), 14.3 (11.6–17.2)) years. All enrolled patients were subjected to anamnestic evaluation, laboratory parameters, JIA subclass, and type of therapy for the disease. A clinical-gnathological evaluation, anamnestic and dysfunctional index (Ai and Di), and magnetic resonance imaging of TMJs were performed in all patients. The test group showed a significant reduction (*p* < 0.001) regarding the clinical findings such as maximal mouth opening, left and rightward laterotrusion and protrusion, and a significant difference in the reported symptoms (TMJ sounds, reduced mouth opening and pain), and Ai and Di (*p* < 0.001) compared to healthy patients. Correlation analysis showed a significant correlation between the median duration of disease and the maximum mouth opening and between visual analogue scale (VAS) score and maximum mouth opening, leftward laterotrusion, rightward laterotrusion, and protrusion. The results obtained in this study suggest that patients with JIA presented a cohort of symptoms in TMJs in comparison with healthy controls. Moreover, a careful TMJs evaluation and an early diagnosis of TMJs dysfunction and regular follow-ups are recommended in order to prevent and reduce functional and chewing problems in patients with JIA.

## 1. Introduction

Juvenile idiopathic arthritis (JIA) includes all chronic forms of arthritis that occur before the age of 16, which lasts over six weeks and cannot be traced back to a known cause [1].

Among the chronic inflammatory diseases of paediatric age, it is the most frequent; its incidence and prevalence range from 2 to 20 per 100,000 children [2]. The female sex is most affected with a ratio of 2:1, with peaks of disease onset detected between 1 and 3 years in females [3].

There are some critical observations in consideration of the pathogenesis and aetiology of JIA. JIA is a chronic disease characterised by T-cell abnormalities, and the pathological features of chronic synovitis suggest possible cell-mediated pathogenesis [4]. Moreover, the JIA may appear as a consequence of an oligogenic or polygenic predisposition. The various forms of the disease show non-mendelian inheritance, a resulting consequence of interactions of multiple genes [5].

Multiple etiologic events (traumas, infections, physical and/or mental stresses, hormonal alterations) have been hypothesised to act on a genetically predisposed subject, which may activate the autoimmune process responsible for JIA [5]. As in periodontal or mucosal damage, the main cytokines involved in cartilage inflammation are tumour necrosis factor (TNF)-α, transforming growth factor (TGF)-β1, interleukin (IL)-1, IL-6, and interferon (IFN)-γ [6]. In particular, cartilage damage appears to be mediated by the production of lytic enzymes that destroy the cartilaginous matrix [7].

The international rheumatology committee (ILAR) reproposed a new classification that has the advantage of being more homogeneous. On the basis of exclusively clinical criteria, different subtypes were distinguished by number and type of joints involved, course, complications, and therapeutic response [8]. According to this classification, JIA was determined, such as:

1. Systemic JIA. The presence of systemic signs typical of the child. The main systemic symptoms are intermittent fever, muscle pains, enlargement/growing of liver, spleen, or lymph nodes volume, pericarditis, and pleurisy [9].
2. Polyarticular JIA. It is characterised by the involvement, in the first 6 months of illness, of 5 or more joints, and it can be distinguished as JIA in two different types: the positive (rare in the child, <5% of all JIA patients) and negative Rheumatoid Factor (RF) forms (30% of all patients with JIA, heterogeneous signs and symptoms, TMJ involvement included) [7,8,9].
3. Oligoarticular JIA. It begins before the age of 16 and lasts for at least 6 weeks. It is characterised by the involvement of fewer than 5 joints (usually big joints) and only 3% of cases show TMJ involvement [10].
4. Psoriatic arthritis.
5. Enthesitis-related arthritis (EAS).

The frequent finding of TMJs involvement in children with JIA is of particular interest. TMJs involvement can be acute during JIA. During the active phase of the disease, the chronicity of inflammation at the joint level can determine the alteration of craniofacial growth (with the development of the following malocclusion), dentofacial developmental anomalies, periodontitis, tooth abrasion, and, finally, a sensitive impairment of patients’ quality life [11,12]. In particular, dentofacial developmental anomalies include small mandible (micrognathia), retrognathic mandible, anterior open bite, and increased craniomandibular angle width [13]. The condylar involvement may change from minimal joint flattening to acute damage of the temporomandibular condyle [14].

Despite these clinical alterations, recent studies have shown that JIA patients that ceased to show signs of active TMJ involvement for at least one year may find benefits from rapid maxillary expansion treatment, since resolving maxillary hypoplasia and impaired dental contacts may allow mandibular repositioning and condylar growth [15,16].

Magnetic resonance imaging (MRI), performed with a closed mouth and an open mouth in sagittal projection and the coronal forecast, is the primary examination in instrumental diagnostics of TMJ [17,18]. The invasive method is represented by arthroscopy that allows articular components direct vision, allowing the possibility to diagnose degenerative or inflammatory processes in TMJs from the first stages, a very limited possibility with computed tomography (CT) or magnetic resonance imaging (MRI) investigations. Arthroscope examination also has therapeutic value as well as diagnostic because it allows the removal of intra-articular catabolites, improving mandibular function and canine guidance and achieving regression of pain symptoms [19,20].

Although TMJ involvement in JIA is a known fact, only a few systematic studies have been carried out on its real incidence and on correlation with clinical-prognostic factors of JIA of any form, both symptomatic and asymptomatic. In the light of these results, the objective of the present study was to better evaluate better the TMJ’s characteristics, morpho-structural alterations, and dysfunctional consequences in a population of patients affected by different forms of JIA.

## 2. Materials and Methods

A total of 51 patients were enrolled from February 2016 to January 2018 in a cohort of a southern Italian population. All patients were informed regarding the study characteristics and gave informed written consent, and were approved by the International Reviewer Board (IRB) of the University of Messina, Italy (17/18). The study was performed according to the Declaration of the World Medical Association in Helsinki guidelines revised in 2000. This study was performed in accordance with STROBE (Strengthening The Reporting of Observational Studies in Epidemiology) guidelines.

### 2.1. Power Sample Size Calculation

The sample size was established considering the mean prevalence of JIA in the population equal to 0.08% as previously shown [1], the study group incidence of 2% (prevalence of JIA in our database), an alpha level of 0.05, and a power of 80%. A minimum sample of at least 24 JIA patients and 25 controls was determined to ensure a good sample size.

In this study, 25 patients (10 males, 15 females) diagnosed with JIA served as a test group, whereas 26 healthy patient respondents to a cross-sectional general health survey (12 males, 14 females) without systemic rheumatic disease and TMJ symptoms served as control group. The controls were matched for age and sex with the JIA group and were randomly selected (Table 1).

The inclusion criteria were (1) diagnosis of JIA (a diagnosis of exclusion, based on familiar and personal anamnesis with particular attention to pain and morning stiffness associated to chronic arthritis lasting >6 weeks, detailed physical examination, serological sampling, and radiological evaluation), (2) at least one sign or symptom of TMJ involvement (i.e., swelling, pain, functional limitation, clicking, mandibular deviation, open bite, and micrognathia), and (3) at least one radiological finding of TMJ involvement through MRI (intraarticular fluid increment, contrast enhancement of mandibular condyle and synovia, and/or degenerative changes of the mandibular condyle and joint structures). Patients who had previously undergone steroid injection and were affected by infections, leukaemia/lymphoma, acute rheumatic fever, Kawasaki syndrome, lupus erythematosus, inflammatory bowel diseases, and without TMJ involvement were excluded from the study.

The sources from which data were collected were medical records and clinical, serological, and radiological investigations. More specifically, the patients involved in the study underwent:

1. An anamnestic and clinical evaluation aimed at defining the type of arthritis, age from disease onset, number of joints involved, uveitis, dactylitis, enthesitis, and the detection of signs and symptoms of the TMJs, such as swelling, pain on mastication, functional limitation, temporomandibular click, and mandibular deviation;
2. Evaluation and dating of the type of therapy (non-steroidal anti-inflammatory drugs (NSAIDs), immunosuppressor drugs, and biological drugs, Tumor necrosis factor (TNF)-α inhibitor) [21];
3. By means of peripheral blood sampling, evaluation of inflammatory laboratory parameters was performed mainly through the analysis of antinuclear antibodies (ANA) titre, erythrocyte sedimentation rate (ESR) and CRP values, and the positivity of rheumatoid factor (RF).
4. TMJ pain/symptoms assessment was performed through a questionnaire (compiled with the help of parents) in which patients had to: (a) report the eventual presence of pain during mouth opening/mastication, morning tiredness/stiffness in the jaws, restriction of the jaw opening, and TMJ sounds; (b) quantify the TMJ-related pain severity though a 100-mm visual analogue scale (VAS) with 0 representing minimum and 10 representing maximum severity, report the pain duration (chronic if ≥ 6 months, non-chronic if < 6 months).
5. Clinical-gnathological evaluation was carried out in which the TMJs physical examination was carried out which consists of the inspection and palpation of TMJs during mouth opening and closing movements [22,23]. With the inspection and the use of a caliper, the degree of maximum interincisal opening (<40 mm was considered reduced, <30 mm was considered a restricted opening [24,25]) and the trajectory of mouth opening was evaluated; furthermore, any click or click-like noises on one or both joints (articular click, popping, or crepitation) or intra-articular click during joint movement were evaluated through a phonendoscope and were assessed by palpation on each side separately [26]. TMJ tenderness was determined through the response to bilateral palpation of the lateral and posterior aspects of the condylar head; masticatory muscle tenderness was evaluated by digital palpation of the temporalis, masseter and pterygoid muscles [27].
6. Upon completion of the clinical-gnathological procedure, patients underwent instrumental evaluation through the X-ray (RX) panoramic and MRI examination of both TMJs. MRI images were used to assess disk morphology and position and to evidence eventual TMJ degenerative bone changes, osteophyte formation, osteosclerosis and/or deformities.

Both groups were evaluated through the Helkimo anamnestic index (Ai) (Ai_0_ no symptom, Ai_I_ mild, Ai_II_ severe symptom) and the dysfunction index (Di) [28] defined as the presence of 5 clinical signs: mandibular mobility, TMJ function, TMJ pain, muscular pain, and pain during movement. The calculated Di score divided patients into 3 groups: Di_0_ (no symptoms), DiI (mild temporomandibular disease [TMD]), Di_II_ (moderate TMD) and Di_III_ (severe TMD).

### 2.2. Statistical Analysis

The Kolmogorov–Smirnov test showed a not normal distribution of the variables; therefore, a non-parametric approach was adopted. The Chi^2^ test was applied referring to the categorical variables to compare JIA and control groups. The relationship analysis between the MRI findings and the mean duration of disease was performed through the Chi^2^ test. With reference to numerical variables, the Mann–Whitney test was applied to compare the groups. In order to assess any significant interdependence between the mean disease duration and VAS vs. the other clinical parameters, the Spearman correlation test was used.

All numerical variables are reported as median and interquartile range (IQR), whereas the categorical data are presented as numbers and percentages. Statistical analyses were performed using SPSS 25.0 statistical software for Windows (SPSS Inc., Chicago, IL, USA). *p*-values < 0.05 were considered statistically significant.

## 3. Results

### 3.1. Descriptive Analysis

All patients completed the study successfully. The median age of the JIA patients was 13.9 (10.9–15.3) years and that of the healthy patients was 14.3 (11.6–17.2) years.

**Figure 1:**
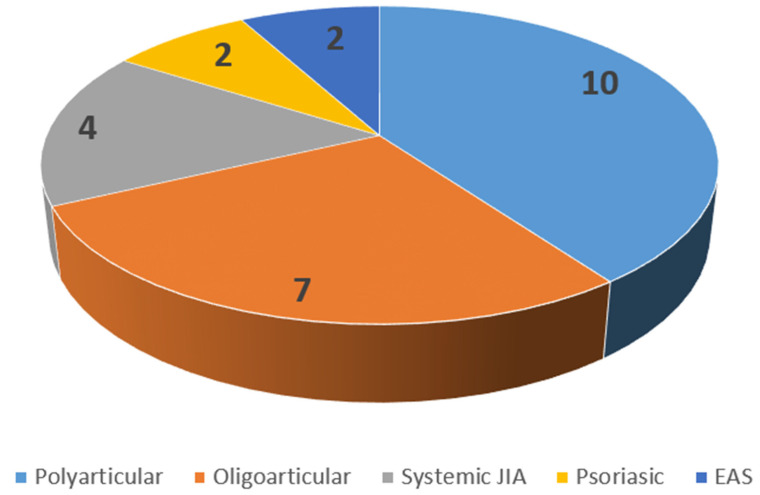
Distribution of JIA forms of the patient sample.

The demographic and clinical characteristics did not show significant differences (*p <* 0.05) among the groups (Table 1). The test group showed a mean JIA onset of 12.2 (11.3–13) years, with TMJs symptoms onset at 12.9 (12.3–13.4) years. The mean duration of the disease in the test group was 5.9 (3.1–9.2) years. The sample was composed of 15 females (60.0%) and 10 males (40.0%), which presented polyarticular forms (10 patients, 40.0%), oligoarticular forms (7 patients, 28.0%), systemic form of JIA (4 patients, 16.0%), psoriatic arthritis (2 patients, 8.0%), and EAS forms (2 patients, 8.0%) (Figure 1). Furthermore, 16.0% (4 patients) of JIA patients presented uveitis.

Regarding drug therapy, 60.0% of patients (9 females, 6 males) undergo a combination therapy (NSAID associated with immunosuppressor drug-methotrexate). Only 24.0% of JIA patients (4 females, 2 males) needed the addition of the biologic drug (Etanercept), while 16.0% of patients (2 females, 2 males) undergo only one type of drug (NSAIDs or methotrexate).

Regarding laboratory data, monitoring of the inflammatory and autoantibody parameters of the JIA group of patients with TMJ involvement documented a clear prevalence of alterations in the female sex, moderately increased ESR values in 68.0% of the examined population. The dosages of CRP and ANA antibodies appeared to be increased by 60.0% and 68.0%, respectively. The positivity of the rheumatoid factor stood out in 80% (20 patients, including 9 males and 11 females) of the JIA subjects.

### 3.2. Clinical Findings and Patient-Reported Symptoms

The JIA group showed statistically significant reduction (*p* < 0.001) regarding the clinical findings such as maximal mouth opening, left and rightward laterotrusion, and protrusion comparing to the control group (Table 2).

Furthermore, regarding the patient-reported symptoms (TMJ sounds, reduced mouth opening, and pain), the comparisons among the two groups showed statistically significant differences (*p* < 0.001). A total of 22 patients (88.0%) in the JIA group reported a reduction of maximum mouth opening, while 21 patients (84.0%) reported the presence of TMJ sounds. Regarding the TMJ-related pain severity, the median VAS score in JIA patients was 8.2 (7–9.1), whereas the healthy patients reported a median value of 2.5 (1.7–3.1) (*p* < 0.001).

A greater number of JIA patients showed at least one clinical finding or symptom compared to the healthy patients (*p* < 0.05).

### 3.3. Clinical-Gnathological Evaluation

**Figure 2:**
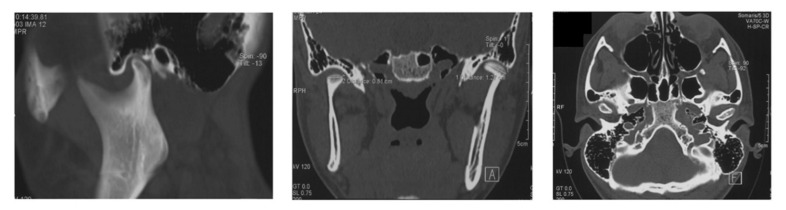
A 7-year-old female patient, affected by oligoarticular form ANA+, RF−, with age of onset 5 years. Joints involved in the onset: right knee and duration of the disease until gnathological evaluation: 9 years in systemic therapy with methotrexate. At the anamnesis, she did not show any previous therapy concerning TMJs and orthodontic treatments. The patient presented a unilateral right tumefaction at the clinical examination, a bilateral temporomandibular click, and the mandibular deviation (to the right).

A higher percentage of JIA group patients showed TMJ dysfunction compared to healthy patients (*p* < 0.001) (Table 3, Figure 2). More specifically, crepitation and clicking were present in 17 and 23 patients, respectively. Furthermore, over 50% JIA patients presented TMJ and muscle tenderness (*p* < 0.05).

### 3.4. Helkimo Anamnestic Index (Ai) and Dysfunction Index (Di)

JIA group patients showed statistically significant Helkimo anamnestic index (Ai) and dysfunction index (Di) score differences compared to the control group (*p* < 0.001) (Table 4). More specifically, 10 JIA patients (40.0%) presented Ai_II_ symptoms, 13 patients (52.0%) Ai_I_ symptoms, and only 2 patients (8.0%) presented Ai_0_. Instead, 1 healthy patient (3.8%) showed Ai_II_ symptoms, 2 patients (7.7%) Ai_I_ symptoms, and 23 patients (88.5%) Ai_0_. Regarding the Di, in the JIA group, 7 patients (28.0%) showed Di_III_ severe signs, 10 patients (40.0%) Di_II_ signs, 7 patients (28.0%) Di_I_ signs, and only 1 patient (4.0%) presented Di_0_ signs. In contrast, in the control group, 0 patients (0%) showed Di_III_ or Di_II_ signs, 3 patients (11.5%) Di_I_ signs, and the remaining 23 patients (88.5%) presented Di_0_ signs.

### 3.5. Correlation Analysis

In JIA patients, correlation analysis showed a significant correlation between the mean duration of disease and the maximum mouth opening (coefficient = −0.418; *p* = 0.032). Furthermore, VAS score was significant correlated to maximum mouth opening (coefficient = −0.698; *p* = 0.000), leftward laterotrusion (coefficient = −0.824; *p* = 0.000), rightward laterotrusion (coefficient = −0.842; *p* = 0.000), and protrusion (coefficient = −0.756; *p* = 0.000). No other significant correlations were found between VAS, the median duration of diseases, and the other clinical variables.

### 3.6. MRI Assessment

**Figure 3:**
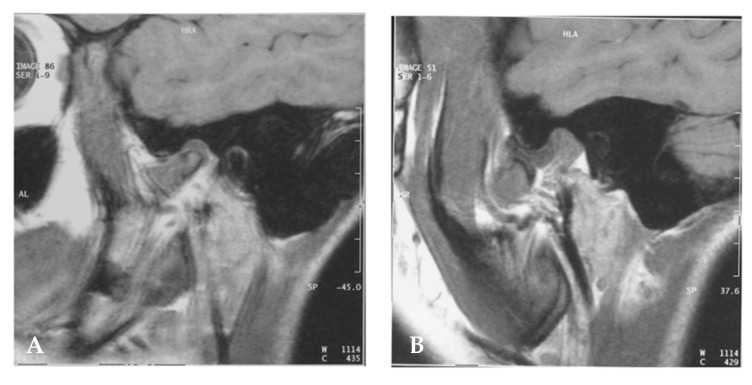
MRI of the right TMJ shows a meniscus which, although thin, is normally positioned between the bony heads with both open mouth (**A**) and closed mouth (**B**). In the closed-mouth study, anterior dislocation of the meniscus is documented with subsequent recapture of the same in the open-mouth study, a finding compatible with a condylar-meniscal incoordination syndrome. The MRI examination also documented a modest hypotrophy of masticatory muscles on the right.

MRI did not show TMJ involvement in the control group. However, the 25 JIA patients showed different TMJ alterations (Table 5, Figure 3): 11 disks without displacement (44.0%), 21 disk displacements with reduction (84.0%), 14 TMJs with degenerative bone changes (56.0%), 10 with condyle anterior surface flattening (40.0%), 12 with joint surfaces erosion and irregularities (48.0%), 11 with flattening of the temporal eminence functional surface (44.0%), 4 with osteophytes (16.0%), and 3 with condyle resorption (12.0%).

## 4. Discussion

The aim of the present study was to analyse the signs and symptoms of TMJ dysfunction in a population of JIA patients compared to healthy subjects. We did not include JIA patients who underwent previous intra-articular corticosteroid injections (IACI) since it was not possible to exclude that these medications could influence mandibular growth and, consequently, the clinical and gnathological parameters detected. In fact, it was shown that IACI induced a reduction in mandibular growth in animals [29]. Furthermore, a systematic review reported that IACI impact on mandibular growth remains an unanswered question; therefore, caution should be applied in growing JIA patients with this medication [30].

The present study demonstrated that JIA patients presented a higher prevalence and severity of TMJs symptoms and signs of dysfunction compared to the control group. TMJ dysfunctions were predominant in patients with polyarticular JIA form, followed by the oligoarticular form. In particular, compared to males, female patients appeared more susceptible to manifest symptoms of TMJs dysfunction (F:M = 2:1). Most of the patients were characterised by active JIA forms with positive inflammatory indexes (such as ANA) and a high Reuma test. Furthermore, the statistical model used in this study showed a correlation between the VAS score reported by the patients and their maximum mouth opening. Therefore, these patients should be instructed to do some exercises to increase mouth opening in order to improve oral hygiene procedures.

JIA is the most frequent autoimmune disease of the paediatric age; during JIA, TMJs can represent the first site of onset and remain one of the main and early affected joints, especially in oligoarticular JIA [31]. In accordance with the present study results, several reports have reported that, in the polyarticular and systemic JIA forms, TMJs can be involved symmetrically and, consequently, JIA patients present difficult mandibular movement excursion [32].

In the present study, JIA patients reported a higher self-reported symptoms rate than the control group. Moreover, the differences reported between self-reported symptoms and respective clinical findings may be explained by the fact that the questions aimed at patients examined past or recent signs, while clinical examination allowed to assess the presence or absence of TMJ symptoms. In contrast, TMJ sounds without relevant VAS scores were reported less frequently in the self-reported questionnaire than the clinical examination observations in the control group. This may be explained because healthy patients do not feel pain during mandibular and TMJ movements and may underestimate the relevance of TMJ sounds. On the other side, this could explain the positive correlation between higher VAS scores and worse clinical TMJ parameters in JIA patients. It is interesting to point out that the present study demonstrated several early TMJs signs of dysfunction (e.g., TMJ click). In fact, several reports have demonstrated that most JIA patients presented, such as patients without JIA but with TMJ dysfunction, in the adult age [33].

Furthermore, at the paediatric age, the inflammation and dysfunction at TMJs have been shown to possibly lead to mandibular condyle alteration with consequent reduced facial growth and micrognathia, craniofacial growth, and the alterations of several oral functions [34], especially during the transient growth phase [35].

It is conceivable that most of the masticatory apparatus-related alterations, including tooth and periodontal abnormalities, are consequences of the extent of mandibular condyle impairment, which is the fundamental centre of the mandibular and maxillary growth, although responsible for inflammation of periarticular tissues [36].

According to the results of this study, other authors have shown, through cephalometric analysis, an impairment of the masticatory apparatus due to altered mandibular growth with consequent reduction of its dimensions and structural characteristics [37,38,39], such as mandibular retroversion, craniomandibular angle obtuseness, variable periodontal alterations, and even mandibular condyle destruction and vertical dimension reduction and maxillary posterior rotation and alteration of the nasal cavities. These alterations are maybe due to a chronic inflammatory state and release of inflammatory mediators, which may determine skeletal malocclusion, dental alterations, and deformation of “bird’s-beak” profile [37].

By MRI assessment, we found a very high rate of disk displacement with reduction and TMJ with degenerative bone changes in JIA patients (Table 4). In cases of condylar-meniscal incoordination syndrome (Figure 3), the abnormal position of the disk may induce alterations in the synovial membrane associated with episodes of insufficient repairing attempts, which can determine condylar bone erosion over time and TMJ arthritis. In fact, TMJ erosion has been reported in other rheumatic diseases [40].

Based on these findings, the data reported in the literature are controversial, given the non-uniformity of the studies, which often occurs considering data and methodologies that cannot be overlapped or compared with each other. However, some authors consider alterations of the mandibular dynamics correlated to an articular involvement, even if the arthritis of the TMJ can hardly be evaluated by clinical examination only. This can help to intercept the patient at risk, but the certainty of the diagnosis always requires a deepening through the execution of an instrumental examination by image [38,39].

## 5. Conclusions

The results of this study evidenced that TMJ involvement is more frequent and severe in JIA patients compared to healthy subjects, especially in female patients with JIA in polyarticular form. The most frequent and severe morpho-structural alterations were found in the polyarticular form; this form is parallelly related to Reuma test positivity, a negative prognostic factor in the evaluation of arthritis severity. Particular attention must be paid, from what emerges from our casuistry, to subjects with polyarticular forms, in which inflammatory indexes do not normalise during therapy and with positive RF.

Moreover, the present results support the hypothesis that an early assessment of TMJs with a proper diagnostic analysis should be suggested in JIA patients at the first signs and symptoms of TMJ involvement. An orthodontic therapy could reduce subjects’ aesthetic and functional problems, often during the puberty phase. The approach to TMJ dysfunction diagnosis and treatment in the context of various forms of JIA requires the collaboration of several specialist figures (paediatrician rheumatologist, radiologist, orthodontist, gnathologist) who interface together in the awareness of this localisation that is poorly contemplated and difficult in the therapeutic approach.

## Figures and Tables

**Table 1:** Demographic and clinical characteristics in the two groups.

Characteristics	Control Group	JIA Group	*p*-Value
Patients (*n* M, *n* F)	(12 M, 14 F)	(10 M, 15 F)	NS
Median age, years (IQR)	14.3 (11.6–17.2)	13.9 (10.9–15.3)	NS
BMI kg/m^2^, median (IQR)	22 (20.9–23.1)	21.9 (21–22.8)	NS
Mean JIA onset, median year (IQR)	-	12.2 (11.3–13)	-
TMJs symptoms onset, median year (IQR)	-	12.9 (12.3–13.4)	-
Overall duration of disease of test group, median year (IQR)	-	5.9 (3.1–9.2)	-
Type of arthritis			
Polyarticular JIA, *n* (%)	-	10 (40.0%)	-
Oligoarticular JIA, *n* (%)	-	7 (28.0%)	-
Systemic JIA, *n* (%)	-	4 (16.0%)	-
Psoriatic arthritis, *n* (%)	-	2 (8.0%)	-
Osteoarthritis, *n* (%)	-	2 (8.0%)	-
Drug therapy			
1 drug (NSAIDs or methotrexate), *n* (%)	-	4 (16.0%)	-
2 drugs (NSAIDs + methotrexate), *n* (%)	-	15 (60.0%)	-
3 drugs (NSAISs + methotrexate + etanercept), *n* (%)	-	6 (24.0%)	-
Laboratory data (*n* increased)			
ANA, *n* (%)	-	17 (68.0%)	-
ESR, *n* (%)	-	17 (68.0%)	-
CRP, *n* (%)	-	15 (60.0%)	-
RF^+^, *n* (%)	-	20 (80%)	-

M: males; F: females; IQR: interquartile range; NS: not significant; BMI: body mass index; NSAIDs: non-steroidal anti-inflammatory drugs; ANA: antinuclear antibodies; ESR: erythrocyte sedimentation rate; CRP: C reactive protein; RF^+^: positivity of rheumatoid factor.

**Table 2:** Clinical findings and reported symptoms in the two groups.

Parameters	Control Group	JIA Group	*p*-Value
Patients (*n*)	*26*	25	-
Clinical findings			
Maximum mouth opening, mm, median (IQR)	44.76 (43.12–46.34)	24.62 (22.39–26.89)	<0.001
Laterotrusion left, mm, median (IQR)	6.84 (6.09–7.43)	2.76 (2.34–3.17)	<0.001
Laterotrusion right, mm, median (IQR)	6.92 (6.24–7.41)	2.84 (2.43–3.21)	<0.001
Protrusion, mm, median (IQR)	4.37 (2.33–6.78)	2.56 (2.18–2.92)	<0.001
Patient reported symptoms			
TMJ sounds, *n* (%)	3 (11.5%)	21 (84.0%)	<0.001
Reduced mouth opening, *n* (%)	4 (15.4%)	22 (88.0%)	<0.001
Pain, (mean VAS score), median (IQR)	2.5 (1.7–3.1)	8.2 (7–9.1)	<0.001
At least one clinical finding, *n* (%)	8 (30.8%)	23 (92.0%)	<0.05
At least one symptom, *n* (%)	7 (26.9%)	24 (96.0%)	<0.05

mm: millimetres; IQR: interquartile range.

**Table 3:** Signs of TMJ dysfunction in the two groups.

Parameters	Control Group	JIA Group	*p*-Value
Patients (*n*)	*26*	25	-
TMJ sounds, *n* (%)	5 (19.2%)	24 (96.0%)	<0.001
Crepitation, *n* (%)	1 (3.8%)	17 (68.0%)	<0.001
Clicking, *n* (%)	2 (7.7%)	23 (92.0%)	<0.001
Popping, *n* (%)	3 (11.5%)	14 (56.0%)	<0.05
TMJ tenderness, *n* (%)	2 (7.7%)	16 (64.0%)	<0.05
Muscle tenderness, *n* (%)	3 (11.5%)	15 (60.0%)	<0.05

**Table 4:** Helkimo anamnestic and clinical dysfunction indexes in the two groups.

Parameters	Control Group	JIA Group	*p*-Value
Patients (*n*)	*26*	25	-
Helkimo Anamnestic Index (Ai)			
Ai_0_ symptoms, *n* (%)	23 (88.5%)	2 (8.0%)	<0.001
Ai_I_ symptoms, *n* (%)	2 (7.7%)	13 (52.0%)	<0.001
Ai_II_ symptoms, *n* (%)	1 (3.8%)	10 (40.0%)	<0.001
Clinical Dysfunctional Index (Di)			
Di_0_ signs, *n* (%)	23 (88.5%)	1 (4.0%)	<0.001
Di_I_ signs, *n* (%)	3 (11.5%)	7 (28.0%)	<0.001
Di_II_ signs, *n* (%)	0 (0%)	10 (40.0%)	<0.001
Di_III_ signs, *n* (%)	0 (0%)	7 (28.0%)	<0.001

**Table 5:** Signs of TMJ dysfunction in the two groups.

Parameters.	Control Group
Patients (*n*)	*26*
Disk without displacement, *n* (%)	11 (44.0%)
Disk displacement with reduction, *n* (%)	21 (84.0%)
TMJ with degenerative bone changes, *n* (%)	14 (56.0%)
Condyle flattening, *n* (%)	10 (40.0%)
Erosions and irregularities, *n* (%)	12 (48.0%)
Temporal eminence flattening, *n* (%)	11 (44.0%)
Osteophytes, *n* (%)	4 (16.0%)
Condyle resorption, *n* (%)	3 (12.0%)

## Data Availability

The data that support the findings of this study are available from the corresponding author upon reasonable request.
